# Plasmonic Nanostructures for Enhanced Self-Healing in Materials: A Review

**DOI:** 10.1021/acsomega.5c03274

**Published:** 2025-09-26

**Authors:** Adriana Nunes dos Santos, Rodrigo J. de Oliveira, Alessandro Chiolerio

**Affiliations:** † Bioinspired Soft Robotics, Center for Converging Technologies, 121451 Istituto Italiano di Tecnologia, Via Morego 30, 16163 Genova, Italy; ‡ Department of Chemistry, State University of Paraiba, Rua Baraúnas 351, Campina Grande 58429-500, Brazil

## Abstract

This work provides a comprehensive analysis of the effects of plasmonic materials on self-healing processes, focusing on recent advances, challenges, and perspectives. We highlight the unique role of plasmonic nanostructures in facilitating the efficient repair of fractures in materials. Despite considerable advances in self-healing materials, there is still great interest in investigating the specific properties and applications of plasmonic nanostructures. In particular, silver and gold nanoparticles exhibit remarkable photophysical properties, attributed to localized surface plasmon resonance, which enables precise control over self-repair processes. This review addresses the physical effects, the fundamental role of thermal energy in healing processes, and the existing challenges, and ultimately outlines future directions for research in the field.

## Graphical abstract

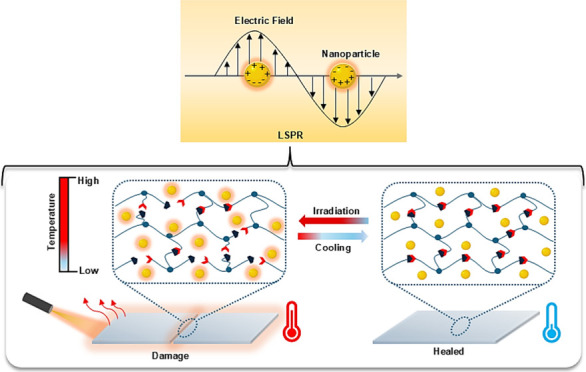

## 1 Introduction

The development of smart materials has gained significant attention in recent decades due to their ability to respond dynamically to environmental stimuli.^1–3^ Self-healing materials have emerged as a new class of smart materials capable of restoring functionality after damage through reversible molecular interactions or the release of embedded healing agents.^4^

The ability to autonomously repair mechanical damage, such as scratches, cracks, or fractures, translates into extended material lifespans, reduced maintenance costs, and increased sustainability.^5^ These benefits have accelerated the adoption of self-healing materials in applications such as soft robotics,^6,7^ wearable devices,^8^ supercapacitors,^9^ sensors,^10^ biomedical devices,^11^ coatings,^12^ automotive,^13^ and e-skins.^14^

Self-healing materials are typically classified into two main categories.^15–20^ First, intrinsic systems rely on reversible physical or chemical interactions (e.g., Diels–Alder reactions,^21^ disulfide bonds,^22^ boronic ester bonds,^23^ hydrogen bonds,^24^ metal–ligand coordination,^25^ van der Waals forces) often enhanced by nanomaterials that reinforce mechanical strength and provide additional dynamic bonding sites.^26^ Second, extrinsic systems employ healing agents stored in microcapsules or vascular networks released upon damage. In these systems, the incorporation of nanoparticles helps to form more robust containers and may accelerate the healing process through improved heat distribution.^27,28^

More recently, the incorporation of plasmonic nanostructures opened avenues for light-triggered self-healing. Plasmonic materials, particularly those based on gold and silver nanoparticles, exhibit localized surface plasmon resonance (LSPR), which enables the efficient conversion of light into localized heat.^29^ This photothermal effect can drive self-repair, such as bond reorganization, melting, recrystallization, or self-assembly at the nanoscale, offering precise spatial and temporal control over healing events.^30,31^

Motivated by these promising characteristics, numerous studies have been conducted to investigate the development of nanocomposites incorporating metal nanoparticles into polymer matrices.^32,33^ The integration of plasmonic nanoparticles into polymer matrices results in plasmonic nanocomposites that exhibit synergistic properties, combining the flexibility and processability of polymers with the optical and thermal capabilities of metallic nanostructures.^34^ These hybrid systems demonstrate improved thermal stability, mechanical robustness, and adaptive functionalities while also enabling controlled nanoparticle dispersion and alignment within the host matrix.^35^ As such, they are gaining attention for applications beyond self-healing that include enhanced electronic,^36–38^ magnetic,^39,40^ mechanical,^41^ optical,^42,43^ and chemical functionalities.^44^

Despite the significant advancements achieved independently in both self-healing materials and plasmonic nanotechnology, their intersection remains relatively underexplored, particularly in the design of next-generation smart materials. This review seeks to address this gap by elucidating the role of plasmonic nanostructures in enhancing self-healing mechanisms with emphasis on recent developments, existing challenges, and future directions. By consolidating current knowledge and shedding light on photothermally driven healing strategies, we aim to stimulate further interdisciplinary research and foster innovation in this rapidly advancing field.

## 2 Plasmonic Self-Healing Materials

Plasmonic self-healing materials restore functionality by leveraging both physical and chemical processes. In various studies, physical healing is primarily driven by localized photothermal effects induced by light irradiation.^45,46^ These effects stem from LSPR, a phenomenon in which electrons in plasmonic nanostructures, such as metal nanoparticles, oscillate collectively when excited by light at specific resonant frequencies.^29,47,48^ This resonance produces a characteristic absorption band in the UV–visible spectrum and generates intense localized heat, which plays a crucial role in damage repair. When light interacts with a plasmonic material, it induces charge oscillations that propagate as an electromagnetic wave along the interface between conductive and nonconductive media. This interaction increases the density of charge carriers, which, when collectively excited, emit radiation in the near-infrared (NIR) region, contributing to heating and enabling material reorganization.^49^

The plasmonic activity of nanoparticles can occur across different spectral ranges depending on their material composition, morphology, and surrounding dielectric environment, enabling flexibility in tuning light–matter interactions for specific self-healing applications. Noble metals such as gold (Au) and silver (Ag) exhibit strong LSPR in the visible to NIR regions, making them widely used in photothermal systems due to their intense absorption, scattering, and chemical stability. Among these, spherical silver nanoparticles produce narrow and intense resonance peaks in the visible region, while gold nanospheres absorb mainly at ∼520 nm. However, anisotropic structures such as gold nanorods, nanoshells, and nanocages can extend their LSPR into the NIR window (∼700–1000 nm).^50^

Although much of the literature has focused on visible and IR-active plasmonic materials, the ultraviolet (UV) spectral range has been exploited in self-healing systems primarily through photochemical mechanisms rather than plasmonic heating. For example, recent works^51^ on UV-curable coatings demonstrate self-healing via photopolymerization and reversible bond formation upon UV irradiation. Similarly, Li et al.^52^ reported UV-triggered healing in microcapsule-containing coatings, though it highlights the challenge of efficiently converting UV light into thermal energy.

Thus, while UV light can drive self-healing through chemical kinetics, integrating UV-active plasmonic nanoparticles remains largely unexplored. Potential candidates include high-plasma-frequency metals, such as aluminum (Al) or magnesium (Mg), which can exhibit LSPR in the UV region. However, challenges, including efficient photothermal conversion under UV, material stability under high-energy irradiation, and limited penetration depth, must be addressed. This suggests a promising yet nascent direction for plasmon-assisted self-healing in the UV domain.^53,54^

In contrast, semiconductor and conductive oxide nanoparticles, including copper chalcogenides (Cu_2–*x*_S, Cu_2–*x*_Se), indium tin oxide (ITO), and titanium nitride (TiN), display LSPR bands red-shifted toward the infrared (IR) region.^55^ Their ability to generate localized heating in the NIR and even mid-infrared ranges expands the scope of plasmon-assisted self-healing strategies, offering new pathways for remote, selective, and efficient damage repair.^56,57^

Plasmonic enhancement in self-healing systems offers significant advantages compared with other self-healing approaches that rely solely on light-absorbing nanoparticles. One key benefit is the ability of plasmonic nanostructures to generate highly localized and efficient photothermal heating at specific wavelengths, enabling precise control over the healing process.^58^ This localized heating minimizes the risk of undesired bulk temperature increases, thereby avoiding damage to the surrounding polymer matrix or sensitive components. Unlike nonplasmonic light absorbers, plasmonic nanoparticles exhibit strong, tunable resonance effects that amplify light absorption and convert it rapidly into heat exactly where it is needed, enhancing the healing efficiency while preserving the overall material integrity.^59–61^

For instance, one report on gold nanoparticles exposed to laser light describes fragmentation followed by agglomeration, a process modulated by zinc phthalocyanines, which helps prevent rapid photoinduced fragmentation.^62^ Similarly, silver-based systems exhibit healing through plasmonic heating.^63^ Other mechanisms involve inhomogeneous electrodynamic interactions in colloidal silver nanoparticles and mechanical responses such as nonlinear stress distribution in 5-fold twinned nanowires.^64^

Chemical healing mechanisms arise from modifications in surface chemistry and interface reconstruction.^63^ For example, in polymer–nanoparticle composites, heat generated by LSPR can induce chemical changes at the interface, triggering effects such as shape-memory behavior and microstructural repair.^65^

Combined physical and chemical processes also contribute significantly to self-healing behavior.^63^ Studies have shown that integrating plasmonic heating with surface or interface modifications via controlled energy input, structural design, or chemical sensitization can produce synergistic effects that enhance material recovery.^61,66,67^ This dual approach offers a versatile framework in which physical phenomena and chemical reactivity cooperate to restore functionality.^60^

The self-healing process in plasmonic materials thus relies heavily on the photothermal conversion via LSPR.^34^ When irradiated with light at the appropriate resonant frequency, plasmonic nanoparticles absorb this light and convert it to localized heat. This temperature rise increases chain mobility in the surrounding polymer matrix, enabling reversible bond dissociation and reformation.^68^ For example, in systems based on polymer, heat facilitates molecular reorganization at the damaged site, and upon cooling, reformation of dynamic bonds restores the material’s integrity (Figure 1). Moreover, the concentration, size, and shape of the plasmonic nanoparticles critically influence the spatial distribution and intensity of the heat generated, thereby affecting the efficiency and extent of the healing process.^48^

**Figure 1:**
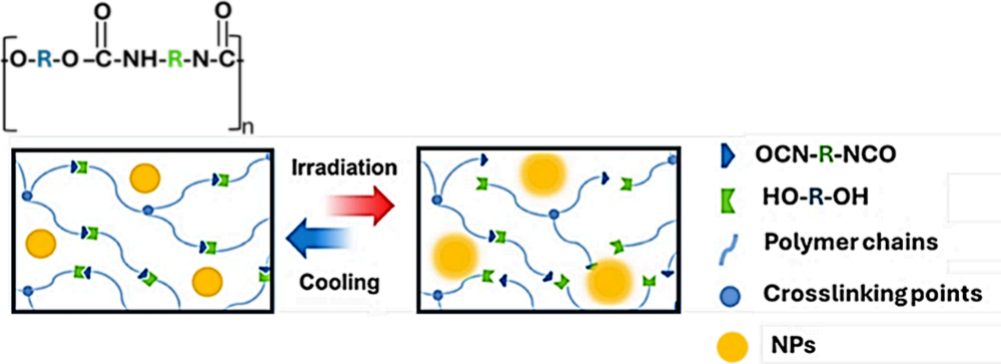
Schematic of the plasmonic self-healing mechanism in a polyurethane matrix.

A recent study conducted by Chen et al.^8^ addressed the development of a plasmonic nanocomposite with excellent light-activated healing capacity. The researchers integrated covalent organic framework (COF) nanosheets with silver nanoparticles (AgNPs) in a polyurethane (PU) matrix (Figure 2a). The resulting material, called AgNPs/TTPA-CONs@PU, is composed of covalent organic nanosheets (CONs) specifically designed from planar 4-connected nodes, TTPA (derived from TAPPDA, N,N,N′,N′-tetrakis­(4-aminophenyl)-1,4-phenylenediamine, and TFPPDA, N,N,N′,N′-tetrakis­(4-formylphenyl)-1,4-phenylenediamine), according to previous studies, and exhibits a rapid temperature increase to 80 °C after exposure to NIR irradiation of 500 mW cm^–2^ for only 12 s and maintained a high optical transmittance of 96.2%. The localized temperature increase allowed a healing efficiency greater than 98% after 4 min of irradiation (Figure 2b).

**Figure 2:**
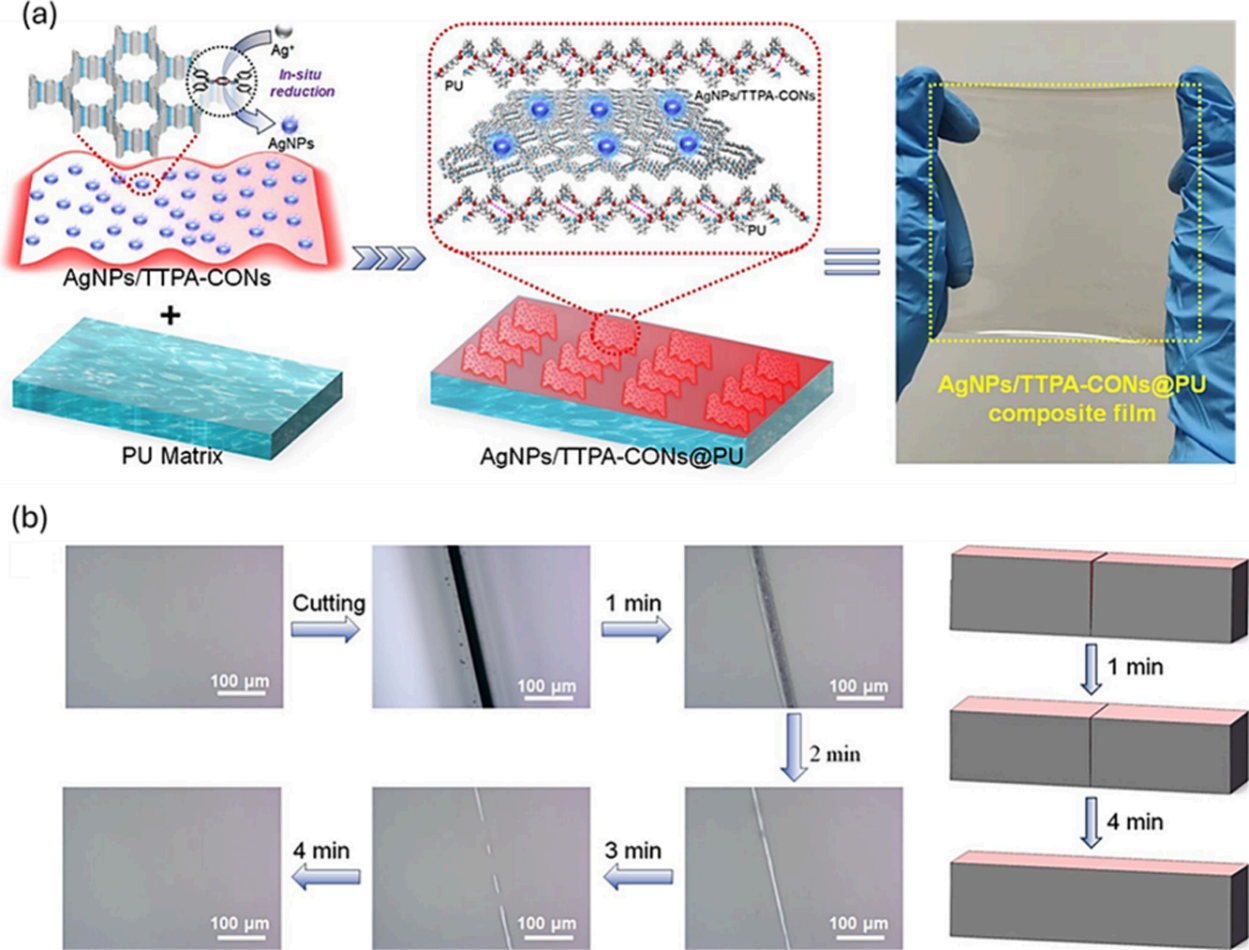
(a) Composite film AgNPs/TTPA-CONs@PU synthesis mechanism. (b) Optical micrographs of the film showing the healing process of damage. Adapted with permission from ref 8. Copyright 2024 ACS.

In this case, the self-healing mechanism was triggered by the heat generated in the AgNPs when irradiated with NIR light. This temperature increase caused the breaking and rearrangement of covalent bonds in the COF structures present in the polymer matrix, allowing the damaged regions to reorganize and form new bonds, thereby restoring the structural integrity of the material. Additionally, as the cracked film was heated, the dynamic chains of the polymer gained mobility, enabling the damaged regions to regain cohesion and restore their structure, optimizing the efficiency of the self-healing process.

In addition to the previously mentioned study, the research conducted by Araya-Hermosilla et al.^56^ investigates the healing capacity of a plasmonic nanocomposite synthesized by the incorporation of ITO nanocrystals (NCs) in a polyketone matrix. In this research, ITO NCs were utilized as an infrared photothermal source due to their remarkable NIR plasmonic properties and visible transparency. The results revealed important self-healing characteristics triggered by NIR irradiation of the nanoparticles, with a remarkable healing time of only 3 s. The self-healing mechanism involves the chemical modification of polyketones with furan groups, which can undergo a reversible Diels–Alder reaction with maleimide compounds. This modification creates a thermoreversible polymeric network capable of curing at high temperatures. The self-healing process is triggered by heat, specifically starting at 50 °C, when the polymer matrix begins to soften. This promotes the reversibility of the Diels–Alder reaction and the decross-linking of the polymer network, which occurs thermally. When the temperature reaches approximately 50 °C, the polymer chains become more mobile, allowing the damaged regions of the matrix to reorganize and self-repair through the reversal of cross-links. The addition of ITO NCs to the polymer network significantly improved the self-healing speed as the ITO nanoparticles enhanced the efficient absorption and conversion of IR radiation into heat, accelerating the local heating process and enabling almost immediate healing. When the temperature reaches 117 °C, the damaged surface heals rapidly, with the crack closing in just a few seconds due to the increased mobility of the polymer chains and the efficiency of the reversible Diels–Alder process. After healing, the material can regain its thermoplastic properties upon cooling, thanks to the reversibility of the thermoreversible network, allowing the material to recover from damage and restore its original properties.

Additionally, nanomaterials such as graphene oxide, carbon nanotubes, and two-dimensional transition-metal carbide/nitride (MXene) exhibit strong IR absorption, enabling the conversion of light into heat.^57,69,70^ These materials have been exploited due to their properties of high mechanical strength, heat conduction capacity, and electrical conductivity. In this regard, incorporating these materials in polymer matrices or other composite materials can result in systems with excellent self-healing ability. For example, Fan et al.^71^ synthesized a transparent and wearable composite coating with optimal light-activated self-healing ability through the hybridization of plasmonic AgNPs, Ti_3_C_2_T_*x*_ nanosheets (MXene), and water-based elastic PU, which was called AgNP@MXene-PU (Figure 3). The study investigated how light can aid crack healing in photothermal coatings. Under the irradiation of 600 mW cm^–2^ to visible and IR light, the researchers observed that the AgNP@MXene-PU composite showed a gradual self-repair ability. It was seen that the cracks in the coatings with only PU did not change after 15 min, while in AgNP@MXene-PU, the damage decreased and disappeared completely in 10 min. Additionally, as the concentration of AgNP@MXene increased in the polymer matrix, the repair process became faster; in these samples, the healing process was completed within approximately 5 min under identical light exposure conditions (Figure 3a). Thermal response played a pivotal role in this study. It was observed that after only 3 min of light exposure, the surface temperature of the materials reached up to 139 °C, allowing fast melting of the polymer matrix and consequently an efficient healing of the damage. Compared with the MXene-PU composite coating, the AgNP@MXene-PU coating produced a higher surface temperature and showed a faster healing process under the same conditions (Figure 3b).

**Figure 3:**
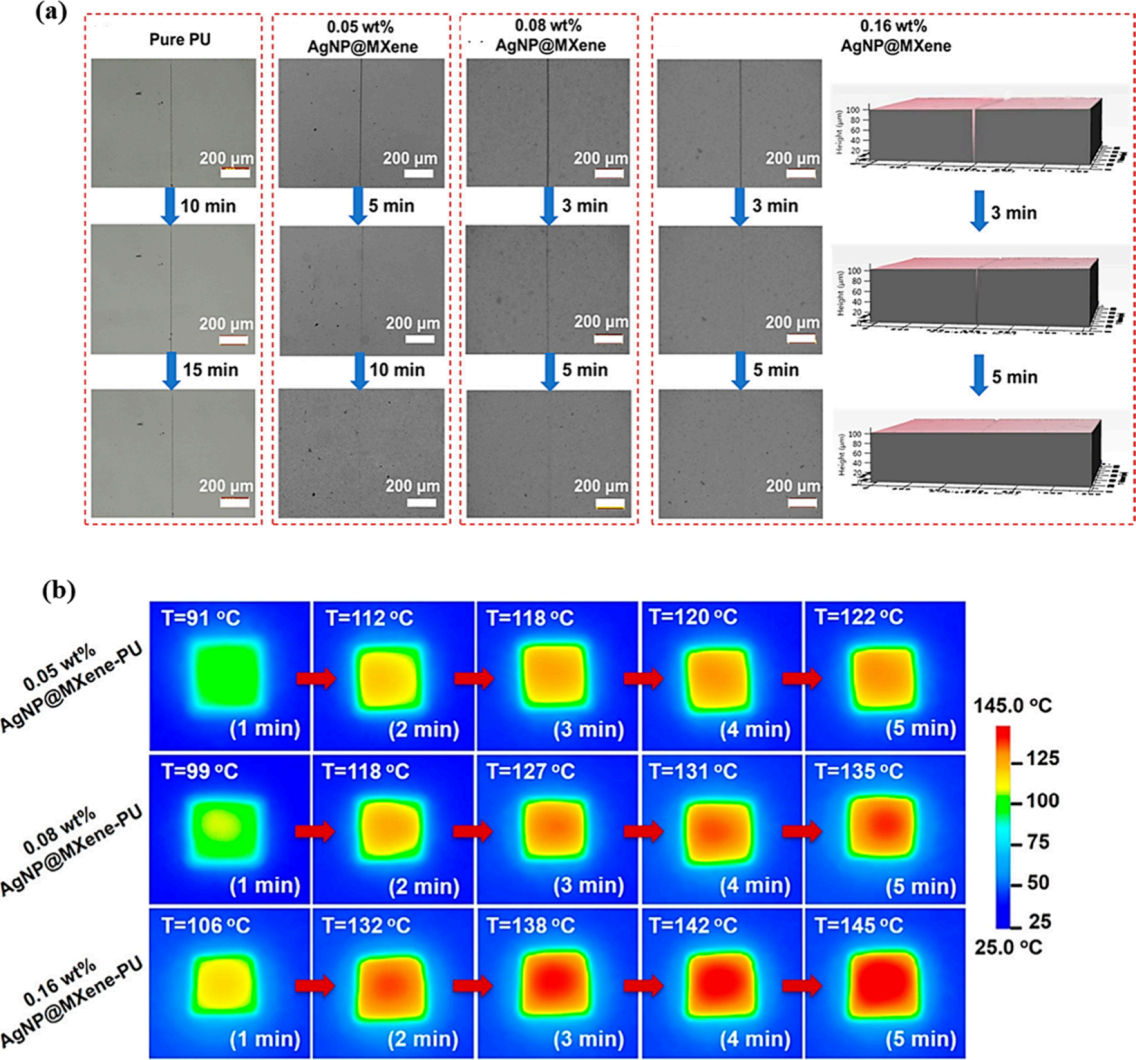
(a) Optical micrographs showing the healing process of the cracked pure PU and 0.05 wt %, 0.08 wt %, and 0.16 wt % AgNP@MXene-PU. (b) Surface temperature of AgNP@MXene-PU coatings with different AgNP@MXene loads was recorded by an infrared thermistor as a function of time under light irradiation of 600 mW cm^–2^ vis-IR. Adapted with permission from ref 71. Copyright 2019 ACS.

AgNP@MXene hybrids in PU show photothermal conversion yielding over 97% healing efficiency, while silver nanowires repair damage via intense pulsed-light or stepped laser power that enhances polymer flowability or bridges nanogaps. The self-healing mechanism of this material involves photothermal conversion generated by the interaction of plasmonic AgNPs with MXene nanosheets integrated into the PU matrix. When the composite is irradiated with vis-IR light, the AgNPs absorb the light and generate heat, amplified by the MXene nanosheets due to their high thermal conductivity. This increase in temperature causes the PU polymer chains to soften, allowing the cracks to close and restoring the material’s integrity. Even with a low concentration of AgNP@MXene (0.08 wt %), the radiation leads to a significant temperature rise (around 111 ± 2.6 °C), sufficient to induce self-healing.

Heat is commonly used as a practical stimulus to initiate reversible cross-linking. Regulating the system’s temperature requires managing the balance between the chemical species involved in the decross-linking process.^72^ Therefore, thermal energy appears as an essential factor influencing the self-healing process, facilitating the effective recovery of materials.^73^

## 3 Role of Thermal Energy in the Self-Healing Ability of Plasmonic Nanomaterials

As previously discussed, the temperature attained by an irradiated plasmonic nanostructure holds significant importance for its role in the self-repair process. Compared to other stimuli, a photothermal self-healing system offers significant advantages, including remote initiation in a highly controlled manner and the ability to instantly switch on and off the light source and direct it specifically to the damaged area.^74^ Table 1 presents the self-repair performance of various plasmonic composites under photothermal activation, highlighting that the self-repair process can be notably efficient despite differences in polymer matrices, irradiation conditions, and nanoparticle types.

**Table 1:** Self-Healing Performance of Plasmonic Composites under Photothermal Activation^a^

composite	self-healing mechanism	wavelength	light intensity	healing time per temperature (°C s)	ref
AgNPs/TTPA-CONs-PU	imine bonds	808 nm	500 mW cm^–2^	19.200	^8^
AgNP-MXene/PU	noncovalent interactions		600 mW cm^–2^	25.020	^71^
Au/PU	carbamate bonds and shape memory	808 nm	5.0 W	10.800	^76^
TiN@mesoporous SiO_2_ core–shell	shape memory	808 nm	2.5 W cm^–2^	2.100	^74^
polyvinyl butyral-Cu_2_O/tannic acid	hydrogen bonds	808 nm	1.5 W cm^–2^	6.900	^75^
ITO/polyketone	Diels–Alder	2250 nm		480	^56^
AuNPs	shape memory	532 nm	80 mW	162.000	^58^
AuNRs		808 nm			
AuNPs/epoxidized soybean oil	covalent bonds	532 nm	700 mW	720.000	^73^
AuNP/poly(N-isopropylacrylamide)	RS-Au bonds	808 nm	1 W	2.700	^77^

a The key performance indicator has been unified by multiplying the healing time by the healing temperature.

An example of this advancement is the NIR-induced self-healing photothermal coatings developed by Cao et al.^75^ In this study, multifunctional nanofibers were prepared with tannic acid, polyvinyl butyral (PVB), and Cu_2_O nanoparticles. It was observed that when NIR irradiation is applied to this composite, the photothermal effect generated by the Cu_2_O plasmonic nanoparticles effectively heats, promoting the healing of cracks in just 100 s. Furthermore, this process allowed for multiple self-healing cycles.

The photothermal effect also enables remote activation in thermoset polymers, characterized by a high glass transition temperature (*T*_g_) or a high degree of cross-linking. For instance, Altuna et al.^73^ synthesized an innovative nanocomposite by incorporating gold nanoparticles (AuNPs) into a biobased polymer matrix. The self-healing mechanism described in this article is based on the photothermal effect of the AuNPs, which are embedded in a polymer network cross-linked with epoxidized soybean oil and citric acid. When the material was irradiated with a green laser, the AuNPs converted the light energy into heat, raising the temperature locally to above 100 °C, thereby enabling the remote activation of self-healing in the cross-linked polymers. This temperature increase triggered the rearrangement of dynamic covalent bonds, particularly β-hydroxyesters, facilitating crack repair.

Plasmonic nanoparticles play a crucial role in the healing process of a polymer involving photothermal effects.^78^ It is important to note that the absorption of light by plasmonic nanostructures, as well as the increase in temperature are strongly dependent on the shape, structure, composition, and wavelength of light. In other words, the wavelength of the LSPR is tunable based on the resonant modes of the structure, which are influenced by geometric parameters such as size, height, width, and depth.^47^

The geometric properties influencing the amplification of the electromagnetic field are the particle dimensions, their distance, and the presence of tips.^55^ By analogy to electrostatics, regions with sharp tips or small radii of curvature can accumulate more charge than regions with smoother surfaces. Consequently, the higher concentration of charges at the tips results in an increased electric field in the nearby region. Therefore, understanding the geometric configuration and material properties of a plasmonic nanoparticle enables the exact calculation of the absorption and scattering areas. This, in turn, facilitates inference of the thermal behavior of the nanoparticle when irradiated with a specific wavelength and beam intensity.^50^

Hot electrons in plasmonic nanoparticles can be generated through multiple excitation mechanisms: primarily via nonradiative decay of LSPR and additionally via intraband transitions in noble metals.^79,80^ The presence of strong localized electromagnetic fields, plasmonic hot spots at interparticle gaps, or sharp structural features (e.g., tips of nanocubes or nanostars^81,82^ ) boosts LSPR-mediated hot electron production, while intraband transitions within the conduction band contribute significantly under suitable excitation, particularly in Au and Ag systems.^80^ As a result, nanoparticle morphology critically influences plasmon resonance properties, hot electron yield, photothermal response, and thus the efficacy of material healing mechanisms.^83^

Manrique-Bedoya et al.^84^ conducted simulations on highly localized heating in Au nanostructures with three different geometries (Figure 4a). The study found that the plasmonic excitation of gold nanorods resulted in a local temperature increase of up to 100 °C, whereas heating using spherical gold nanoparticles reached only 37 °C. Similarly, Chen et al.^85^ observed that gold nanoparticles with thorn geometry (Figure 4b) exhibited significantly higher solar absorption efficiency compared to spherical gold nanostructures.

**Figure 4:**
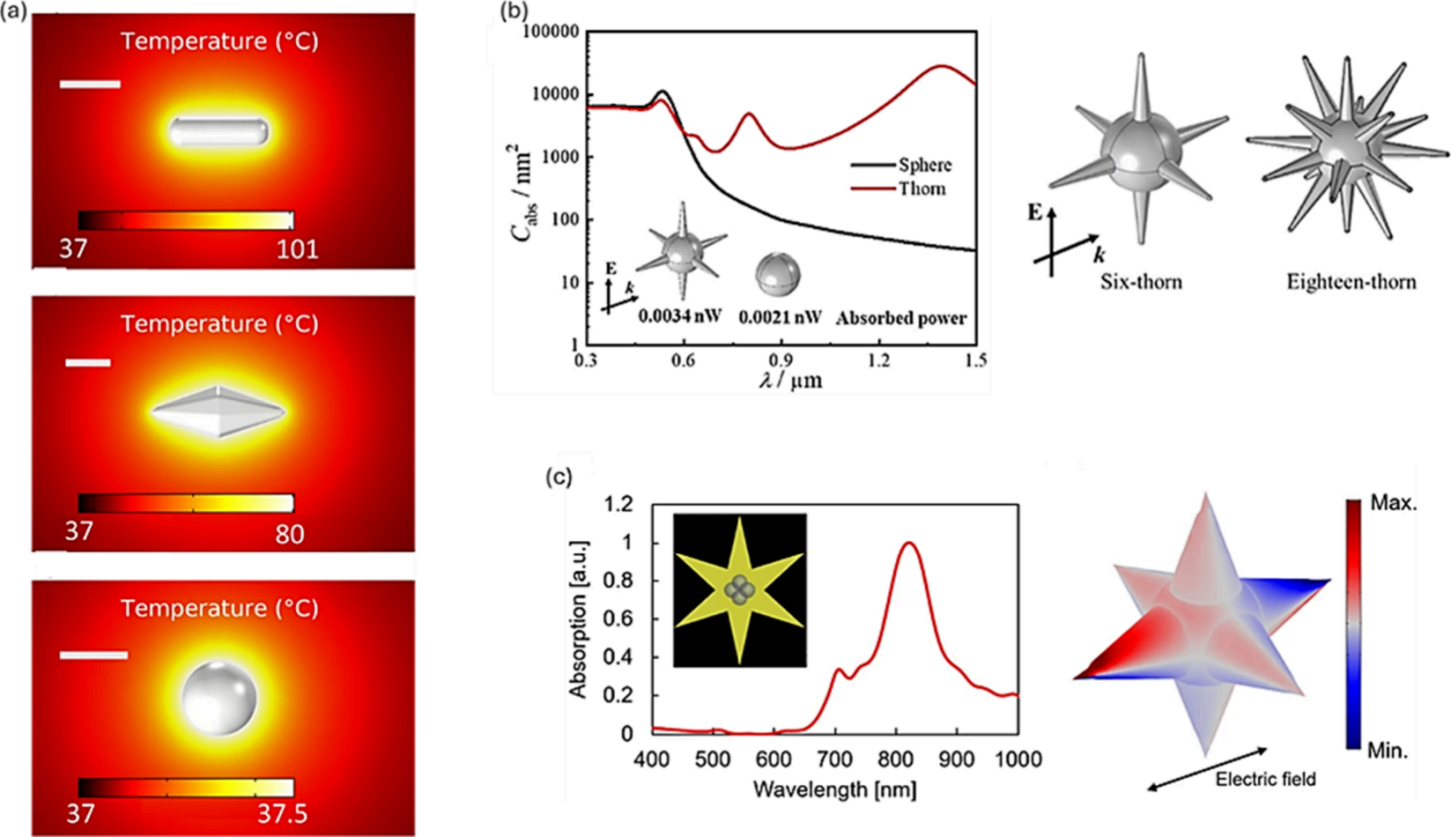
(a) Steady-state temperature maps of the nanorod, nanopyramide, and sphere. Scale bars are 50 nm. Reprinted with permission from ref 84. Copyright 2020 ACS. (b) Absorption cross-sections for nanoparticles of the thorn and Au sphere, respectively. Adapted with permission from ref 85. Copyright 2020 Elsevier. (c) Absorption spectra simulated by FEM of plasma nanostars and the charge distribution in the resonance wavelength. Reprinted from ref 87. Copyright 2020 Springer Nature.

In small nanoparticles, the electromagnetic field within them is uniform. However, as these particles grow, multipolar plasmon resonance occurs, as seen in thorn nanoparticles and nanostar geometries.^86^ This complexity of light interaction with such particles increases with their size. For example, Tomitaka et al.^87^ employed finite element method (FEM) simulations to explore NIR-activated star-shaped gold plasmonic nanostructures (Figure 4c). The findings revealed a single plasmon resonance with multipolar mode excitation. Rodríguez-Oliveros and Sánchez-Gil^88^ elucidated the temperature profile of gold nanoparticles with this geometry under optical heating. Their study showed that star-shaped nanoparticles, particularly those composed of Au, serve as excellent candidates for efficient heat sources. The results indicated that these nanostructures exhibited a higher heating efficiency compared to their equivalent spherical counterparts, suggesting that the star geometry contributed to enhanced energy absorption and heat generation. Moreover, an increase in the number and sharpness of the nanostar tips resulted in a higher thermal efficiency. The symmetry of the nanoparticles was also investigated, revealing that more symmetrical structures led to a substantial increase in the generated temperature.

These studies suggest that plasmonic nanoparticles with more complex shapes offer valuable insights into the thermal properties of these nanostructures, emphasizing their potential to significantly contribute to the development of new self-healing materials and technologies. The utilization of plasmonic nanostructures as heat sources is currently being explored for biomedical applications including drug delivery and tumor therapy. When combined with mechanically soft materials such as elastomers, these composites present new possibilities for enhancing various multifunctional materials.^57,89^

### 3.1 Fundamentals of Plasmonic Heating

Plasmonic heating is a physical phenomenon in which plasmonic nanostructures absorb incident light and convert the electromagnetic energy into heat, which is subsequently dissipated in the surrounding environment. This process is highly dependent on several parameters, including the shape and material composition of the nanostructures, as well as the wavelength of the incoming light.^41^ The underlying mechanism begins with the excitation of localized surface plasmons, where conduction electrons in metallic nanoparticles oscillate collectively in resonance with the incident electromagnetic field. At specific frequencies, this interaction leads to a phenomenon known as LSPR, which significantly amplifies the local electromagnetic field around the nanostructure.^50^

The enhancement of electron oscillations at resonance results in frequent collisions between electrons and the atomic lattice, a process termed Joule heating. This energy dissipation mechanism is primarily responsible for converting light into thermal energy in plasmonic systems.^69^ Efficient thermoplasmonic heating occurs when the incident light frequency matches the LSPR frequency of the nanostructure, maximizing the absorption and conversion of energy into heat. One of the most important parameters for evaluating plasmonic heating is the absorption cross-section, which indicates how effectively a nanostructure can absorb light at a given wavelength. The greater the absorption cross-section, the higher the potential thermal power that can be generated, making it a key indicator of photothermal efficiency.^90^

The temperature distribution resulting from plasmonic heating can be modeled using the heat-transfer equation, incorporating properties such as density, specific heat capacity, thermal conductivity of the surrounding medium, and power generated via Joule heating. Typically, a steady-state temperature is achieved within tens of nanoseconds following irradiation.^50^ The geometry of the nanostructure plays a decisive role in defining its optical response and heating efficiency. Parameters such as size, shape, and composition determine the LSPR frequency and influence how efficiently light is absorbed. For example, spherical gold nanoparticles display LSPR in the UV–visible region with increasing particle size leading to a redshift and broadening of the absorption band.^85^ More complex geometries, such as gold nanoshells and “nanomatryoshkas” (core–shell structures with metal–insulator–metal architecture), can be engineered to absorb in the NIR region by adjusting parameters, such as the core diameter and shell thickness. Gold nanorods, which possess both longitudinal and transverse plasmon modes, offer another tunable system. The longitudinal mode can be precisely tuned across a wide range of the spectrum by changing the aspect ratio of the nanorod.^30,84,91^ The shape and size of Ag nanoparticles also influence the absorption spectra in the visible range. Ab initio simulations confirm that a spherical geometry results in a remarkable redshift of the first absorption peak from 382 to 406 nm when its size increases from 38 atoms to 140-atom clusters.^92^ Furthermore, a much larger redshift occurs, changing the shape (aspect ratio) of the nanoparticles; in particular, an aspect ratio variation of 93% shifts the first absorption peak from 382 to 450 nm. Changing the shape of a nanoparticle (i.e., its aspect ratio) and keeping the number of atoms fixed has a much more pronounced effect on the absorption spectra when compared to a change in size.

Another important consideration is the thermal stability of the plasmonic nanostructures.^93^ These materials are often subjected to high temperatures during irradiation, which may lead to morphological changes that compromise their optical and plasmonic properties. For instance, gold nanorods are known to reshape at temperatures significantly lower than the melting point of bulk gold, which can lead to a loss of their photothermal functionality. Moreover, the surrounding environment plays a crucial role in heat dissipation.^61^ Factors such as the thermal conductivity of the surrounding medium, the presence of interfaces (e.g., glass–water boundaries), and the formation of vapor layers around the nanostructure can alter both the absorption efficiency and the resulting temperature profile.

The shape of the plasmonic nanostructures significantly influences their optical and thermal properties, allowing for tailored photothermal responses for specific applications. Gold nanospheres, for example, typically resonate in the visible range, and their absorption properties change with size.^84^ While larger spheres shift the resonance to longer wavelengths, smaller spheres tend to absorb more efficiently at their respective resonant frequencies when normalized by the projected area. Other materials, such as titanium nitride and platinum, offer absorption in the NIR region, presenting alternative options for applications requiring deeper light penetration. Gold nanoshells, composed of a dielectric core (such as silica) and a thin gold shell, are another versatile platform.^87^ Their plasmon resonance can be shifted into the NIR region by modifying the ratio of core to shell, enabling efficient light-to-heat conversion with relatively small particle sizes.^75^

Gold “nanomatryoshkas,” which consist of a gold core, a dielectric spacer, and an outer gold shell, provide further control over the optical response. By adjustment of the thickness of each layer, these multilayered structures can be designed to exhibit strong NIR absorption while maintaining diameters below 100 nm, making them suitable for biomedical applications, such as in vivo photothermal therapy. Gold nanocages, with their hollow and porous structures, also display tunable LSPR in the NIR region, depending on the wall thickness and porosity. Their unique architecture makes them particularly attractive for applications involving controlled drug release in addition to efficient photothermal conversion. Overall, the precise control of geometry and composition allows for the optimization of plasmonic heating across a wide range of disciplines, including nanomedicine, catalysis, and soft robotics.^29^

## 4 Applications of Plasmonic Self-Healing Materials

The design of systems that combine plasmonic and self-healing properties represents an emerging innovation in materials science and nanotechnology. The applications of plasmonic self-healing materials are expected to grow significantly due to the integration of different classes of plasmonic materials in addition to the promising advantages they can offer to various technological areas.

To date, Ag and Au nanoparticles are the most widely used nanomaterials in the advancement of these self-healing materials. This poses economic challenges as noble metals feature unstable markets and high costs. Nevertheless, the volumes of self-healing materials are not high as they represent a specialty and not a commodity, and their added value is high; therefore, they can be sold at higher prices.

Last, other plasmonic materials are available, including titanium nitride (TiN) and other transition-metal nitrides, which have recently attracted considerable attention due to their favorable plasmonic properties in the NIR region, high thermal and chemical stabilities, and biocompatibility.^56,94^ Compared with Au and Ag nanoparticles, TiN offers a more cost-effective and robust option, making it a promising candidate for practical plasmonic self-healing systems. Whose cost is much lower: for example, a range of prices for colloidal Au is between 50 and 200 USD per mL, while colloidal TiN ranges between 5 and 30 USD per mL. Current self-healing polymers market estimates are in the range of 500 MUSD with a CAGR of around 5% (as of 2024).

Prominently, promising alternatives to conventional noble metals are being explored in the field of plasmonic materials. For example, metal oxides exhibit flexible plasmonic properties and are particularly favored by their low cost, which can accelerate their practical implementation.^71^ Another innovative strategy involves using quantum dots that can be exploited as plasmonic materials. Studies have shown that incorporating quantum dots into polymeric systems can simultaneously enhance the materials’ photothermal conversion, mechanical properties, and self-healing performance.^95^ The synergy between quantum dots and the polymer matrix improves the efficiency of photothermal processes due to the high light absorption capacity of quantum dots and reinforces the structural integrity of the material, promoting a more effective response to damage and enabling self-repair. Thus, the integration of quantum dots into plasmonic self-healing materials presents a promising path for creating advanced and multifunctional systems, enhancing both the durability and functionality of nanomaterials.

Another promising approach to developing plasmonic self-healing materials involves the use of semiconducting polymer particles as plasmonic agents. Studies show that semiconducting polymers can undergo chemical modifications that confer optical properties similar to those of noble metals.^96,97^ This feature paves the way for the use of conductive polymers in the modulation of plasmonic responses. For example, nanodisks of a highly conductive polymer such as poly (3,4-ethylenedioxythiophene:sulfate) can act as dynamic plasmonic nanoantennas, eliminating the need for metallic nanostructures. The polymer itself takes on the role of plasmonic material due to its high mobility and density of polaronic charge carriers. A remarkable feature of these nanoantennas is their ability to be completely turned on/off through chemical adjustments to the redox state of the polymer, resulting in significant changes in the conductivity and optical properties of the material.^96^ This ability to adjust is very promising for the self-healing process of plasmonic materials as it allows precise control over the plasmonic response in specific regions of the material.

Although plasmonic self-healing materials are still a relatively new field of research, they already show potential for a variety of applications (summarized in Table 2), including sensors, electronic devices, catalysts, photocatalysts, nanomedicine, energy conversion, coatings, electrodes, and wearable devices.

**Table 2:** Plasmonic Self-Healing Material Applications from 2018 to 2024

plasmonic material	matrix	application	plasmonic and self-healing properties	year	ref.
AgNWs	PU	flexible electronic	irradiation with intense-pulsed-light	2018	^66^
			high electrical conductivity		
AuNRsAuNPs	polyacrylamide hydrogels	nanomedicine	reversible shape transitions (rigid-low rigid) under ON/OFF irradiation	2019	^58^
			shape memory		
			selective wavelength for self-healing (532 nm for NPs and 808 nm for NRs)		
AuNRs	PU vitrimer	electronic devices	laser radiation (808 nm, 1 W, 70 to 180 °C at 5.0 W	2019	^76^
			shape memory		
AgNPs/Ti_3_C_2_T_*x*_/MXene	PU	wearable device	600 mW cm^–2^ results in 111 °C in 5 min	2019	^71^
			healing efficiency >97%		
			synergistic effect of MXene and AgNPs		
FFTNWs	bilayer graphene	nanoelectronics devices	3.07 × 10^19^ W/m^3^, λ = 392 nm	2020	^64^
TiNNPs	epoxy	coatings	UV–Vis-NIR absorption	2021	^74^
			shape memory		
ITONPs	furan grafted polyketone	soft robotics	NIR irradiation	2022	^56^
			strong absorption between 1500 and 2250 nm with temperatures >160 °C within 30 s		
Cu_2_ONPs	polyvinyl butyral and tannic acid	coatings	NIR absorption	2022	^75^
			fast healing in 100 s		
AgNPs	PU	wearable devices	500 mW cm^–2^ NIR light irradiation results in 80 °C at 12 s	2024	^8^
			healing efficiency >98%		
			synergistic effect between AgNPs – PU sheets		

As discussed in this review, one of the key properties of plasmonic materials is their ability to generate localized heat via the photothermal effect. This unique feature offers numerous advantages in the field of nanomedicine. By exploitation of their strong light-matter interactions, plasmonic nanomaterials can be used for a variety of therapeutic and diagnostic purposes. For instance, their capacity to produce spatially confined heating enables targeted photothermal therapies, selectively destroying cancer cells while minimizing damage to surrounding healthy tissues.^90^ Furthermore, plasmonic nanostructures can be functionalized with therapeutic payloads, such as drugs, peptides, or genetic materials, allowing for site-specific delivery and enhanced treatment efficacy with reduced systemic toxicity.^67,98^ Their role also extends to advanced imaging techniques, including magnetic resonance imaging and positron emission tomography, enabling real-time monitoring and improved diagnostic precision.^99^ In addition, the versatility of plasmonic materials facilitates their integration into multifunctional platforms such as biosensors^100^ and stimuli-responsive drug-delivery systems.^60^

Despite the promising performance of plasmonic self-healing systems, several critical challenges must be addressed for their safe and effective use, especially in biomedical applications. First, material degradation under repeated light exposure can alter the optical properties and reduce the photothermal efficiency over time. For instance, studies on gold nanorod aggregates have demonstrated changes in absorption profiles after multiple heating–cooling cycles, which can affect their photothermal stability.^101^ Second, toxicological risks are well documented: biodistribution studies reveal that AuNPs accumulate predominantly in the liver and spleen, with toxicity strongly influenced by the particle size, shape, surface chemistry, and coating.^102^ Specifically, anisotropic shapes like nanorods and nanotriangles often induce higher cytotoxicity compared to spherical nanoparticles,^103^ and small AuNPs (< 30 nm) can generate reactive oxygen species, leading to oxidative stress and DNA damage.^102^

Moreover, the long-term thermal and structural stabilities of plasmonic composites during repeated self-healing cycles remain underexplored. Although short-term studies on gold nanorod aggregates have demonstrated stable photothermal behavior over a week of laser cycling, comprehensive evidence of performance retention in cyclic healing contexts is lacking.^104^ These limitations underscore the need for further research into biocompatible, degradable, and stable plasmonic systems as well as improved understanding of their interactions with host materials and biological environments. Additionally, scalability and reproducibility remain significant challenges in the development of plasmonic nanomaterials. Variability in nanoparticle synthesis, particularly in terms of size, shape, and surface chemistry, can lead to inconsistent optical properties and photothermal performance when integrated into composite systems. Such batch-to-batch variations hinder standardization and limit the translational potential of these materials for practical applications.

For example, the study conducted by Wang and his collaborators^58^ made significant advances in developing plasmonic self-healing materials by integrating Au nanoparticles (λ_max_ = 530 nm) or Au nanorods (λ_max_ = 790 nm) into two DNA-based hydrogel matrices. The presence of Au nanoparticles allowed the occurrence of controlled morphological transitions in the hydrogel. That is, when subjected to ON/OFF irradiation, the hydrogels demonstrated reversible transitions between deformed states of low rigidity and rigid states in the form of a triangle. To investigate the self-healing property, the rigid hydrogels were irradiated with a 532 nm (80 mW) laser, which caused the transition to low stiffness states. The resulting hydrogels were cut into two pieces and physically connected under the same laser source. When the light source was turned off and the hydrogels cooled, the matrices were integrated and completely self-healing. The results also show that the self-healing efficiency varied according to the concentration of the AuNPs. In this case, higher concentrations of the nanoparticles resulted in materials with a higher rigidity and better mechanical properties after the healing process.

It is worth noting that in addition to the functions of shape memory and self-healing, hydrogels showed the ability to release drugs in a controlled way. The irradiation of the hydrogels favored the rapid release of drugs (such as doxorubicin), with activation/deactivation (ON/OFF) of the light source controlling the release process. On the other hand, hydrogels without nanoparticles showed inefficient release.

Plasmonic materials have also been explored as anticorrosion coatings, yielding promising results. For instance, Cao and his collaborators^75^ developed three-dimensional core–shell nanofibers with plasmonic self-healing properties. They utilized Cu_2_O nanoparticles for photothermal conversion, tannic acid as a healing agent, and poly­(vinyl butyral) as a polymer matrix. The healing process occurred within 100 s, facilitated by the mobility of the polymer chain after the Cu_2_O nanoparticles efficiently converted light into heat under NIR laser irradiation (808 nm, 1.5 W cm^–2^). This study demonstrated that the combination of intrinsic healing activated by NIR irradiation and extrinsic healing induced by healing agents offers an efficient and innovative solution for corrosion protection in marine engineering.

Another research conducted by Ma et al.^74^ presents results of new plasmonic coatings with self-healing properties activated by the photothermal response of titanium nitride (TiN) nanoparticles. In this study, mesoporous TiN@SiO_2_ core–shell nanocontainers were created to support benzotriazole (BTA) corrosion inhibitors, which were incorporated into an epoxy coating matrix with shape memory. To investigate the self-healing effect, the material was irradiated under a NIR laser (808 nm, 2.5 W cm^–2^). The heat generated enabled the release of BTA from the nanocontainers in the damaged regions while simultaneously activating the shape memory effect of the coating. The healing process began in just 30 s of NIR illumination. This plasmonic self-healing material approach is very promising for the development of coatings with high efficiency and fast healing times.

Another important field being explored is devices and soft robotics, where plasmonic self-healing materials introduce novel approaches to sensing and actuation while simultaneously driving the development of new materials and architectures. All of these applications underscore the advantages of plasmonic self-healing. Specifically, their ability to respond to external stimuli, such as light and temperature, underscores the versatility of these materials across various technology domains, fostering innovation in diverse sectors.

## 5 Future Outlook

This review highlights innovative approaches for utilizing plasmonic nanostructures for the self-healing process. These plasmonic materials exhibit photophysical properties, such as LSPR, which facilitate the efficient conversion of light into heat. Therefore, this mechanism has been exploited to induce self-repair in the damaged materials.

We observed that the integration of nanoparticles into elastomer polymers has broadened the scope of applications for these materials. Thus, coatings and films based on plasmonic nanocomposites offer several advantages, including the ability to respond to external stimuli, such as light and heat, thereby promoting an effective self-repair process. It is noteworthy that the generation of hot electrons through unconventional geometries, such as nanostars and thorn-shaped structures, has shown potential for enhancing the quantum efficiency and improving the thermal properties in plasmonic materials. Among these geometries, the star-like gold nanoparticles emerge as one of the most studied on the nanometer scale, just behind the gold nanorods, due to their inertness, responsiveness to light, and biocompatibility. These nanostars have an adjustable optical response, particularly in the vis-NIR region, which is controlled by the size of the core and the number and length of the branches.^86^ This characteristic, combined with the intense confinement of light at their ends, makes them of great interest, especially for various biological applications.^105^

Although current research continues to explore plasmonic materials based on noble metals such as Au and Ag and has made significant progress, they still face several challenges. One of the problems is the high cost and limited availability of gold and silver, which restrict their large-scale applicability. To overcome this issue, a promising approach is to investigate the recycling of electronic waste to produce plasmonic nanoparticles. Gold is one of the most valuable metals present in e-waste. Gold concentrations are estimated to range from 300 to 350 g/ton in mobile phones and from 200 to 250 g/ton in printed circuit boards,^106^ while the average concentration in rocks commonly ranges between 0.5 and 5 mg/ton.^107^ In this regard, utilizing electronic waste as a source of metals for synthesizing high-value nanomaterials, such as plasmonic gold nanoparticles, presents an innovative strategy for advancing nanotechnologies. This approach not only mitigates environmental concerns associated with electronics disposal but also reduces costs related to the acquisition of precious metals. Moreover, it offers an environmentally sustainable solution for obtaining these materials, promoting a circular economy and sustainability within the advanced technology industry.

Another interesting strategy to boost the viability of plasmonic self-healing materials on a large scale is to explore additive manufacturing, which opens a wide range of possibilities. In this perspective, 3D printing has emerged as a tool that allows the creation of objects by melt processing or by photocuring, offering a cost-effective and scalable way. The advantages of 3D printing also include the possibility of manufacturing structures with complex geometries that would be unfeasible to obtain with other methods, as well as allowing customized production according to the needs of each application.^42^ Elastomeric self-healing materials must be designed to provide thermomechanical features that allow their use in additive manufacturing, in this sense.

From this perspective, we believe that the exploration of plasmonic self-healing materials can revolutionize several fields, opening the way to a wide range of innovative applications. As shown in this review, recent studies have highlighted the versatility of these materials applied in various areas, ranging from sensors and catalysis to electronic devices, electrodes, and wearable devices, as well as the promising field of nanomedicine. The latter, although still underexplored using plasmonic self-healing materials, is attracting considerable interest. In addition to their healing properties, plasmonic self-repair materials offer significant advantages in biological applications. For example, plasmonic self-healing materials can be used in the development of controlled drug-release systems, allowing precise administration of therapeutic agents at the target site, minimizing side effects, and improving treatment efficacy.^58^ Furthermore, their ability to adjust and regenerate can be exploited to create adaptable biomedical devices that conform to the patient’s physiological conditions, providing a personalized and optimized therapeutic response.

Another promising application lies in tissue engineering and organ regeneration, where plasmonic self-healing materials can be used to develop biomimetic scaffolds that promote cell growth and tissue regeneration. These materials can be designed to dynamically adapt to changes in the biological environment, facilitating the integration and functionality of regenerated tissues.^108^ In addition, their ability to respond to external stimuli, such as light or temperature, makes them ideal for image-guided therapy applications, allowing noninvasive monitoring of the healing and regeneration process.

Therefore, continued progress in the research and development of plasmonic self-healing materials is essential to unlocking their full potential and exploring new horizons in nanotechnology. The joint integration of optical, magnetic, and electrical characteristics, coupled with the attributes of self-repair in engineered materials, will undoubtedly drive the course of future technological innovations.
